# Isolation and Identification of Endophytic Fungi from *Syzygium cumini* Linn and Investigation of Their Pharmacological Activities

**DOI:** 10.1155/2022/9529665

**Published:** 2022-11-03

**Authors:** Mst. Mabiya Sultana Samapti, Farhana Afroz, Satyajit Roy Rony, Suriya Sharmin, Fatema Moni, Shammi Akhter, Sheikh Feroz Uddin Ahmed, Md. Hossain Sohrab

**Affiliations:** ^1^Pharmaceutical Sciences Research Division, BCSIR Laboratories, Dhaka, Bangladesh Council of Scientific and Industrial Research, Dhaka-1205, Bangladesh; ^2^Department of Pharmacy, Jahangirnagar University, Savar, Dhaka 1342, Bangladesh

## Abstract

This study was conducted to isolate and identify the endophytic fungi from the bark and leaves of the *Syzygum cumini* plant and investigate the pharmacological activities of endophytic fungi along with plant parts. After isolation, endophytic fungi were identified based on morphological characteristics and molecular identification. Antimicrobial, antioxidant, and cytotoxic activities were studied by a disc diffusion method, free radical scavenging DPPH assay, and brine shrimp lethality bioassay, respectively. A total of eight endophytic fungi were isolated and identified up to the genus level based on morphological characteristics and confirmed by molecular identification techniques. Among the eight isolates, three isolates were identified as *Colletotrichum* sp. (SCBE-2, SCBE-7, and SCLE-9), while the rest of the isolates belonged to *Diaporthe* sp. (SCBE-1), *Pestalotiopsis* sp. (SCBE-3), *Penicillium* sp. (SCBE-4), *Phyllosistica* sp. (SCLE-7), and *Fusarium* sp. (SCLE-8). The presence of flavonoids, anthraquinones, coumarins, and isocoumarins was assumed by the preliminary screening of the fungal and plant extracts by a thin-layer chromatographic technique under UV light. Fungal extracts of *Pestalotiopsis* sp. *Penicillium* sp. were found sensitive to all test bacteria, but only extracts from the leaf and bark showed significant antifungal activity along with their antimicrobial activity. *Penicillium* sp. The fungal extract showed the highest free radical scavenging activity (2.43 *μ*g/mL) near that of ascorbic acid (2.42 *μ*g/mL). Some fungal extracts showed cytotoxic activity that, in general, suggests their probable abundance of biological metabolites. This is the first approach to investigate the endophytic fungi of *Syzygium cumini* Linn. in Bangladesh, to find the pharmacological potential of endophytes, and to explore novel compounds from those endophytes.

## 1. Introduction

Mankind always rely on nature, especially when it comes to find new drugs to cure diseases [1]. The need for safe, chemical free, and less toxic treatment options has prompted the search for innovative and enhanced ways to deal with these health-related problems. To serve this purpose, several medicines including antioxidants, antidiabetic drugs, anti-inflammatory agents, antiulcer agents, antibiotics, and antifungal agents have been discovered from different sources like medicinal plants, edible and medicinal higher fungi (mushrooms), microbial sources like fungus, bacteria, and endophytes [2–6]. Recently, many known as well as new endophytic bioactive metabolites have been identified as possessing a wide variety of pharmacological activities such as antibiotic, antiviral, anticancer, anti-inflammatory, and antioxidant [7]. So, endophytes associated with plants may be considered a source of novel compounds [8]. Endophytes are microbes that are present in internal plant tissues without inducing symptomatic infection in their host plant. Endophytic fungi have contributed to their host plants by developing bioactive compounds that provide protection to the plant [9, 10]. In each plant, one or more endophytes can reside, so plants provide a rich microorganism repository [11, 12]. In this study, *Syzygium cumini* has been used for investigation. *Syzygium cumini* (Syn. *Eugenia jambolana*), also known as jamun or black plum, belongs to the family *Myrtaceae* [13]. The entire plant parts such as seed, fruit, leaves, flower, and bark are widely used in folk medicine like Ayurveda, Unani [14, 15]. Studies have shown that various extracts of jamun exhibits pharmacological effects such as anti-inflammatory, antifungal, antiviral, anti-inflammatory, antiulcerogenic, cardioprotective, antiallergic, anticancer, radio protective, antioxidant, and hepatoprotective properties [8]. So, endophytes residing in *Syzygium cumini* may contain a lot of biologically active metabolites. Therefore, the present study was designed to investigate the pharmacological potential of endophytic fungi isolated from the *Syzygium cumini* plant to explore some novel drug candidates as well as to compare them with the plant extract.

## 2. Materials and Methods

### 2.1. Collection of Plant Material and Isolation of Endophytes

The plant sample *Syzygium cumini* Linn was collected from the Jahangirnagar University botanical garden in November, 2018. It was identified by the taxonomist of the Bangladesh National Herbarium, Dhaka, Bangladesh. A voucher specimen (DACB-47653) has been deposited in the Herbarium for further reference. Leaves and barks from healthy and mature plants were randomly collected for the study. The plant material was carried to the laboratory in sterile bags and processed within a few hours after sampling. They were then cut with an anti-cutter, over a sterile glass plate. Endophytic fungi were isolated from the fresh plant parts following the procedure and suitably modified [16–18].

### 2.2. Morphological Identification of Endophytic Fungus

To identify the isolates of endophytic fungus, slides prepared from cultures were stained with a lactophenol cotton blue reagent and then examined with a bright-field and phase contrast microscope [19]. Their morphological identification was completed based on the growth pattern, hyphae, the color of the colony and medium, surface texture, margin character, aerial mycelium, sporulation and production of acervuli, the coloration of the medium, and the size and coloration of the conidia using standard identification manuals [20].

### 2.3. Molecular Identification

The molecular identification of fungal strains was completed by DNA amplification and sequencing of the internal transcribed spacer (ITS) region using the molecular biological protocol [21, 22]. A segment of fungal hyphae (0.5–1.0 cm^2^) was collected from the Petri dish and lyophilized in an Eppendorf tube (2 mL) (Eppendorf, Germany). The lyophilized fungal mycelia were pulverized and the infection was disrupted. Fungal DNA isolation was achieved according to the manufacturer's protocol by using the DNeasy Plant Mini Package (QIAgen, USA). The procedures include cell lysis, RNA digestion by RNase A, removal of precipitates and cell waste, DNA shearing, precipitation, and purification. Then, the isolated DNA was amplified by polymerase chain reaction (PCR). The PCR was performed using the Master Mix Kit of HotStarTaq (QIAgen, USA). As primers, ITS 1 (with TCCGTAGGTGAACCTGCGG base sequences) and ITS4 (with TCCTCCGCTTATTGATGATGC base sequences) (Invitrogen, USA) were mixed with the HotStarTaq Master Mix Kit and DNA template with a total volume of 50 *μ*L. The mixture was then added to the thermal cycler using the programmed PCR (BioRad, USA). The amplified fungal DNA (PCR product) was then submitted by a commercial service for sequencing and the base sequence was compared using BLAST Algorithm with publicly accessible databases, including GenBank.

### 2.4. Endophytic Fungal Cultivation and Extraction

Small scale cultivation was performed on Petri dishes for the isolated fungal strains with approximately 1 L of PDA media for each strain. Endophytic isolates were incubated for 21 days at 28°C and the culture media were extracted two times with ethyl acetate to obtain the crude extracts [17]. The extracts of the fungi were concentrated into a solid residue by evaporation with a rotary vacuum evaporator under reduced pressure to obtain an ethyl acetate crude extract [23].

### 2.5. Preliminary Chemical Analysis

All the extracts, including fungal and plant, were qualitatively tested for the presence of chemical constituents. The screening of extracts was performed by visual detection, UV light (both in short at 254 nm and in long at 365 nm), and vanillin-H_2_SO_4_ spray reagent [17].

### 2.6. Antioxidant Activity

The antioxidant activity of extracts was estimated using the 1,1-diphenyl-2-picrylhydrazyl (DPPH) radical scavenging method. 2.0 mL of methanol solution of the extract at different concentrations was mixed with 2.0 mL of a DPPH methanol solution (20 *μ*g/mL). Samples were placed in a dark place at room temperature for 30 min to complete the reaction. Then, the absorbance was measured at 517 nm against methanol as blank by a UV spectrophotometer using the method described by Brand Williams [24]. For each sample, the result was presented as an IC_50_. Ascorbic acid (AA) and 2-tert-butyl-4-hydroxyanisole (BHA) were used as positive controls.

Inhibition of free radical DPPH in percent (*I*%) was calculated as follows:

(1) $$\begin{array}{c} \left(I\%\right)=\left(1-\frac{A_{\text{sample}}}{A_{\text{control}}}\right)\times 100\text{.} \end{array}$$

### 2.7. Antimicrobial Activity Test

Antibacterial and antifungal activities of the fungus and plant parts were analyzed by using a slightly modified disc diffusion method using four pathogenic bacterial strains, namely, *Staphylococcus aureus* ATCC 25923, *Escherichia coli* ATCC25922, *Bacillus megaterium* ATCC 13578, and *Pseudomonas aeruginosa* ATCC 27833 and one fungal strain, *Aspergillus Niger* [25]. Kanamycin sensitivity disc (30 *μ*g/disc) was used as a positive control for bacteria, whereas ketoconazole disc (30 *μ*g/disc) for fungi. Solvents are used as a negative control. The zones of growth inhibition around the discs were measured after 18 to 24 hours of incubation at 37°C for bacteria and 48 to 96 hours of incubation at 28°C for fungi. The sensitivity of the test agent to the microorganisms is determined by measuring the diameter of the zone of inhibition expressed in millimeters.

### 2.8. Brine Shrimp Lethality Bioassay

The eggs of brine shrimp nauplii (*Artemia salina*) were commercially collected from a pet shop for the brine shrimp lethality bioassay, hatched for 24 hours, and tested for LC_50_ values against varying concentrations (200–0.39 *μ*g/mL) of dimethylsulfoxide (DMSO) fungal extracts obtained by the serial dilution technique [26]. Vincristine sulfate was used as a positive control and DMSO was used both as a solvent and as a negative control. All the samples were tested in triplicate.

## 3. Results

### 3.1. Morphological Identification of Fungal Isolates

**Figure 1:**
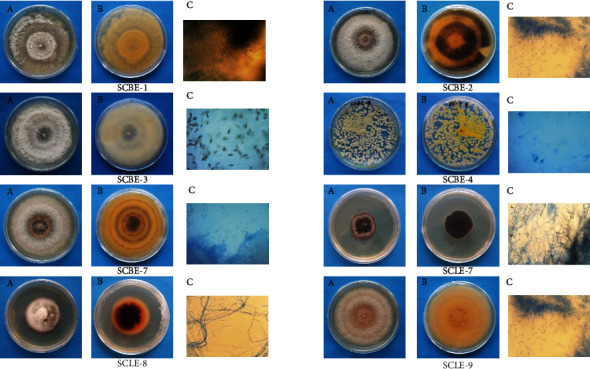
Morphological identification of isolated fungi. (a) Front view, (b) back view, and (c) microscopic view.

A total of eight endophytic fungi were isolated from the bark and leaf parts of the plant *Syzygium cumini*. They have been coded as internal strain numbers, namely, SCBE-1, SCBE-2, SCBE-3, SCBE-4, SCBE-7, SCLE-7, SCLE-8, and SCLE-9. The microscopic characters of strain SCBE-1 produced woolly to cottony, whitish, pale to light brown mycelium, conidiophores that were simple. Two types of conidia were observed upon microscopic examination. The alpha (*α*) conidia were hyaline, fusiform to ovate, straight, aseptate, and frequently biguttulate. The beta (*β*) conidia were filiform, sigmoidal, and hyalinase arising from the mycelium. These characteristics indicated that the fungus SCBE-1 belongs to *Diaporthe* sp. (Figure 1). Strains SCBE-2, SCBE-7, and SCLE-9 showed profusely branched, septate, and hyaline mycelium. Simple, elongated, disc-shaped, or cushion-shaped conidiophores were also found. Conidia were hyaline, single-celled, and ovoid to oblong in shape. This fungus developed quickly, usually covering the entire Petri dish in 10 days and sporulation was abundant on PDA media. These characteristics indicated that the fungi belong to *Colletotrichum* sp. (Figure 1). Their morphology was different and supposed to belong to different species. Strain SCBE-3 was characterized as *Pestalotia* sp. for the presence of white colour mycelium and dark, discoid, or cushion-shaped acervuli. Conidiophores (annellides) were present within tight fruiting structures (aecervuli or pycnidia). Spores (conidia) were 4- to 5-celled, with two or three dark brown center cells (Figure 1). Strain SCBE-4 showed hyaline, fluffy, colored, branched, and septate mycelium. Conidia was arising singly or less often in synnemata from the mycelium, branched near the apex to form a brash like, ending in phialides, eventually turned into green and granular spores, one celled, ovoid. This characteristic indicated that the fungus SCBE-4 belongs to *Penicillium* sp. (Figure 1). In the case of SCLE-7, pycnidia were dark, ostilate, lenticular to globose, which were immersed in the host tissue. Short or obsolete conidiophores were observed. Conidia were small, 1-celled, hyaline, ovoid to elongated. So, SCLE-7 was characterized as *Phyllosistica* sp. (Figure 1). SCLE-8 produced sparse to abundant cottony mycelium with white violet mycelium and pigmentation from colorless to pale violet. Conidia or phialo spore's hyaline, two kinds of spores were observed. Macroconidia were multicellular organisms with a pointed end that was slightly curled or bent. Microconidia were one-celled and oblong in shape, with a fluffy colony and spores that were cylindrical, septate, and aseptate. So, strain SCLE-8 was identified as *Fusarium sp*. (Figure 1).

### 3.2. Molecular Identification of Fungal Isolates

The identification of isolated strains SCBE-1, SCBE-2, SCBE-3, SCBE-4, SCBE-7, SCLE-7, SCLE-8, and SCLE-9 was further confirmed by DNA sequence analysis based on mega BLAST software at the U. S. National Centre for Biotechnology Information (NCBI). Among the eight isolates, three isolates were identified as *Colletotrichum* sp. (SCBE-2, SCBE-7, and SCLE-9) while the rest of the isolates were *Diaporthe* sp. (SCBE-1), *Pestalotiopsis* sp. (SCBE-3), *Penicillium* sp. (SCBE-4), *Phyllosistica* sp. (SCLE-7), and *Fusarium* sp. (SCLE-8).

**Figure 2:**
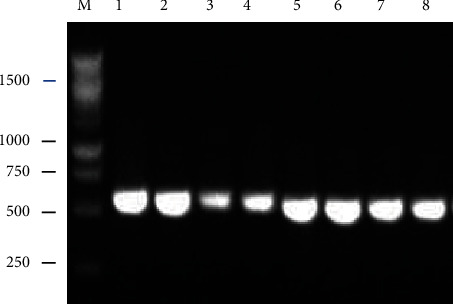
The visualization of amplification PCR (100 volts, 30 mins). (M) marker, (1) SCBE-1 strain, (2) SCBE-2 strain, (3) SCBE-3 strain, (4) SCBE-4 strain, (5) SCBE-7 strain, (6) SCLE-7 strain, (7) SCLE-8 strain, and (8) SCLE-9 strain.

The high-quality DNA extracted from endophytic fungi used ITS1 and ITS4 as primers for PCR. The genomic DNA of the fungi that successfully amplified SCBE-1 and SCBE-2 isolates had a length of 625 bp; the SCBE-3 and SCBE-4 isolates had a length of 600 bp, while the SCBE-7, SCLE-7, SCLE-8, and SCLE-9 isolate had a length of 550 bp (Figure 2). Their detailed description of GenBank accession number of closely related fungal strains, along with their similarity and family is summarized in Table 1.

### 3.3. Preliminary Screening

A thin-layer chromatographic technique was conducted to determine the presence of various secondary metabolites. The screening of extracts was performed by visual detection, UV light (both in short at 254 nm and in long at 365 nm), and vanillin-H_2_SO_4_ spray reagent and presented in a tabular form (Table 2). This result indicates the presence of a good number of compounds like flavonoids, terpenoids, steroids, coumarins, isocoumarins, anthraquinone, etc., which are depicted in a tabular form.

### 3.4. Antimicrobial Screening of Endophytic Fungi from *Syzygium cumini*

For screening of antibacterial activity, all the extracts obtained from endophytic fungi and the plant samples were tested at 100 *μ*g/disc concentration. The zone of inhibition produced by the fungal and plant extracts was found to be 7–22 mm. Leaf extract of *Syzygium cumini* plant exhibited significant antimicrobial activity against *Escherichia coli* (18 mm), good activity against *Salmonella typhi* (15 mm), moderate activity against *Bacillus megaterium* (13 mm), and poor activity against *Staphylococcus aureus* (7 mm). It demonstrated significant antifungal activity against *Aspergillus niger* (22 mm), which is higher than the standard ketoconazole (18 mm). The bark extract also showed mild activity against *S*. *typhi* (10 mm). On the other hand, its zone of inhibition against *A*. *Niger* was 18 mm. The extract of *Penicillium* sp. (SCBE-4) was found to be sensitive to all test bacteria, exhibiting good activity against *E*. *coli* (15 mm), *S*. *typhi* (16 mm), *S*. *aureus* (15 mm) and mild activity against *B*. *megaterium* (10 mm). Also, an extract of *Pestalotiopsis* sp. (SCBE-3) was found sensitive to some test bacteria, exhibiting mild activity against *E*. *coli* (10 mm), poor activity against *S*. *typhi* (8 mm), *S*. *aureus* (7 mm), and *B*.*megaterium* (7 mm). Another fungal strain *Colletotrichum* sp. (SCLE-9) showed poor activity against *S*. *aureus* (7 mm). On the other hand, all the test bacteria were found resistant to the strain *Diaporthe* sp. (SCBE-1), *Colletotrichum* sp. (SCBE-2) (SCBE-7), and *Phyllosistica sp*. (SCLE-7). Overall, leaves of *Penicillium* sp. showed significant antimicrobial activity than others fungal extracts. The result of the antimicrobial screening has been presented in the following Table 3.

Extracts from the plant (bark and leaf extract) and fungal strains of SCBE-3, SCBE-4, and SCLE-9 showed antimicrobial activity which indicates that they may produce secondary metabolites with antimicrobial potential.

### 3.5. Antioxidant Activity of Plant and Fungal Extracts

**Figure 3:**
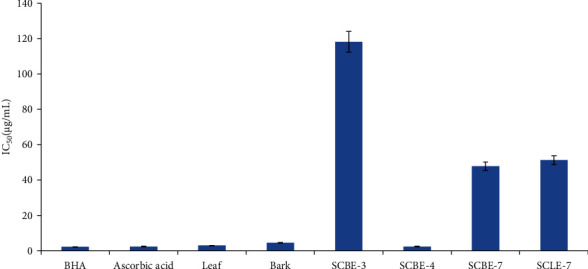
Antioxidant activity of plant and different fungal extracts.

In the free radical scavenging assay, the extract of the leaf and bark part of the plant showed significant antioxidant activity with an IC_50_ value of 2.93 and 4.42 *μ*g/mL, respectively. Comparatively, the leaf extract of the plant exhibited better antioxidant activity than the bark extract. Among the endophytic fungi, *Penicillium* sp. (SCBE-4) showed the highest free radical scavenging activity which the IC_50_ value (2.43 *μ*g/mL) was near to that of ascorbic acid (2.42 *μ*g/mL) and butylated hydroxy anisole (BHA) (2.14 *μ*g/mL). It was also impressive that SCBE-4 showed more antioxidant activity than the leaf and bark parts of the plant. Another fungus *Fusarium* sp. (SCLE-8), *Colletotrichum* sp. (SCBE-7), and *Phyllosistica* sp. (SCLE-7) exhibited antioxidant activity with IC_50_ values of 42.99, 47.77, and 51.23 *μ*g/mL respectively. However, the IC_50_ value was not possible to determine in case of the rest of the fungi because they have not exhibited 50% free radical scavenging activity (Figure 3).

### 3.6. Brine Shrimp Lethality Bioassay

**Figure 4:**
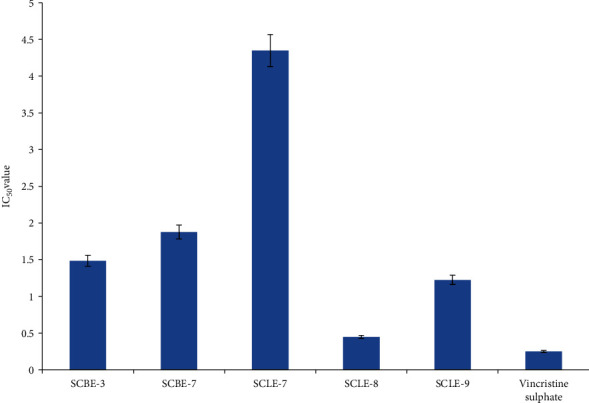
Cytotoxic activity of plant and different fungal extracts.

In the brine shrimp lethality assay, the most potent activity was found from *Fusarium* sp. SCLE-8 (LC_50_ 0.4467 *μ*g/mL) and least activity from *Phyllosistica* sp. SCLE-7 (LC_50_ 4.3481 *μ*g/mL). Other strain of *Colletotrichum* sp. (SCLE-9), *Pestalotiopsis* sp. (SCBE-3), and *Colletotrichum* sp. (SCBE-7) exhibited a LC_50_ value of 1.2259, 1.4848, and 1.8776 *μ*g/mL, respectively. However, LC_50_ could not be determined in case of leaf, bark, *Diaporthe* sp., *Colletotrichum* (SCBE-2), and *Penicillium* sp because their extract did not show 50% mortality in any concentration (Figure 4). So overall, the results suggest that extracts of *Fusarium* sp. (SCLE-8), *Colletotrichum* sp. (SCLE-9), *Pestalotiopsis* sp. (SCBE-3), and *Colletotrichum* sp. (SCBE-7) may contain secondary metabolites having antitumor activities.

## 4. Discussion

The need for discovering new chemical enteritis to treat different ailments is ever increasing as the world is continually facing new threats like cancer, AIDS, COVID-19 etc. Furthermore, the development of resistance of antibiotics and anticancer drugs is a global concern. From the ancient time, plants have served as a source of potential bioactive compounds against different ailments. Recently, endophytes associated with plants earned much attention from the researchers as they are offering metabolites with higher therapeutic potential than plants themselves [27]. Endophytic fungi are amazing organisms colonizing inside the plant tissue and are established as reservoirs of excellent bioactive metabolites [28]. Thus, the screening of bioactivities of natural products, either medicinal plants or fungi, with the aim to discover potential lead molecules can be underestimated.

Isolation of eight endophytic fungi from this plant was reported here, namely, *Colletotrichum* sp. (SCBE-2, SCBE-7, SCLE-9) while the rest of the isolates were belongs *Diaporthe sp*. (SCBE-1), *Pestalotiopsis* sp. (SCBE-3), *Penicillium* sp. (SCBE-4), *Phyllosistica* sp. (SCLE-7), and *Fusarium* sp. (SCLE-8). *Diaporthe* species is known as a major plant pathogen causing infections [29]. However, it has been reported to exert potential bioactivity [30]. *Colletotrichum* sp. is a widely distributed fungal genus reported as plant pathogens, but these fungi have been reported to produce secondary metabolites with diverse bioactivities [31]. Besides these, *Colletotrichum* sp. has been reported as a novel endophytic fungus which is able to produce taxol with excellent cytotoxic activity [32, 33]. *Penicillium herquei*, an endophytic fungus isolated from *Cordyceps sinensis*, produced three new *α*-pyrone derivatives [34]. *Pestalotiopsis microspora* isolated from *Terminalia morobensis* was enriched to the antioxidant compounds pestacin and isopestacin [11, 35]. *Phyllosticta capitalensis* is an endophyte and weak plant pathogen with a worldwide distribution presently known from 70 plant families [36]. Fusarium wilt is one of the major diseases caused by *Fusarium oxysporum* pathogenic strains. Some *F*. *oxysporum* strains are actually beneficial to the host and can provide protection against root pathogens [37]. Therefore, the goal of this study was to look into the endophytic fungi of *Syzygium cumini* as a source of bioactive compounds with cytotoxic, antioxidant, and antimicrobial properties. Preliminary screening of fungal extracts of isolated fungi reveals the presence of flavonoids, coumarin, isocoumarin, steroids, and many other bioactive phytochemicals.

Extracts of the endophytic fungal isolates and bark and leave extracts were subjected to screen for preliminary antimicrobial study by the disc diffusion method, cytotoxicity using the brine shrimp lethality bioassay, and antioxidant activity by DPPH free radical scavenging activity.

Overall extracts of leaf and *Pestalotiopsis* sp. and *Penicillium* sp. strain showed significant antimicrobial activity against tested human pathogenic bacteria *S*. *typhi*, *B*. *megaterium*, *E*. *coli*, and *S*. *aureus*. Only extracts of bark and leaves showed antifungal activity. The bioactivity screening of fungal crude extracts reveals the presence of strong and specific antimicrobial activity against different bacteria and fungi. Specific bioactivity may be defined as high inhibition of the growth of one type of target organism with little or no activity against others. This phenomenon suggests the presence of compounds that have specific modes of action as opposed to highly toxic compounds that are often of little use as therapeutics, which is interesting in drug discovery [38].

In the free radical scavenging assay, comparatively, the leaf extract of the plant exhibited better antioxidant activity than the bark extract. Among the isolated endophytic fungi, *Penicillium* sp. showed the highest free radical scavenging activity (IC_50_ of 2.43 *μ*g/mL, near to the IC_50_ value of ascorbic acid 2.42 *μ*g/mL) than any other fungal and plant extracts. So, it may be stated that *Penicllium* sp. may be a prominent source of the novel antimicrobial and antioxidant compound. In the brine shrimp lethality assay, the most potent activity was found from *Fusarium* sp. (LC_50_ of 0.4467 *μ*g/mL) and the least activity from *Phyllosistica* sp. (LC_50_ of 4.3481 *μ*g/mL). These results are consistent with other reports [35, 39–42].

Furthermore, phytochemical screening of the leaf, bark, and fungal extracts reveals the presence of several bioactive metabolites like flavonoids, terpenoids, steroids, coumrins, isocoumarins, anthraquinone, etc. Those classes of phytochemicals are found to exert several pharmacological activities like antibacterial, cytotoxic, antioxidant activity, etc. This finding is in line with the findings of previous researchers who found a variety of bioactive compounds in endophytic fungal extracts and medicinal plants [43–45].

## 5. Conclusions

This study is an attempt to isolate the endophytic fungus from the *Syzygium cumini* plant and explore the pharmacological activities of those fungi along with the plant part. This study showed that this plant is a plethora of endophytes that are capable of exerting many antioxidants and antimicrobial as well as cytotoxic activities. Plant parts are also bioactive. Thus, this approach may be a way of acquiring novel metabolites having a diverse range of biological activities. However, these endophytic fungi may contain biologically active compounds. Further research can be conducted to isolate the compound and investigate its pharmacological activities.

## Figures and Tables

**Table 1:** NCBI blast results of the fungal isolates recovered from *Syzygium cumini*.

Isolates code	Closely related species	Families of the isolated fungi	Identity (%)	Sequence ID
SCBE-1	*Diaporthe arecae*	*Diaporthaceae*	99.3	KC3430321
SCBE-2	*Colletotrichum gloeosporioides*	*Glomerellaceae*	99.64	MH863850
SCBE-3	*Pestalotiopsis microspore*	*Amphisphaeriaceae*	96.35	MH863951.1
SCBE-4	*Penicillium herquei*	*Trichocomaceae*	99.66	MH864239.1
SCBE-7	*Colletotrichum gloeosporioides*	*Glomerellaceae*	95.48	MH863850.1
SCLE-7	*Phyllosistica capitalensis*	*Botryosphaeriaceae*	100	MF380925.1
SCLE-8	*Fusarium oxysporum*	*Nectriaceae*	98.33	MK380924.1
SCLE-9	*Colletotrichum siamens*	*Glomerellaceae*	100	MK693214.1

**Table 2:** Preliminary chemical screening of fungal isolates and plant extracts.

Test sample	Visual color	UV light at 254 nm	UV light at 365 nm	After spray	Remarks on probable compounds
*Diaporthe* sp. (SCBE-1)	Orange	Light purple	Brown	Purple	Anthraquinone
	Brown	Light purple	Blue	Green	Coumarin
		Purple	Sky blue		Isocoumarin
		Brown			Steroid
		Purple			

*Colletotrichum* sp. (SCBE-2)	Orange	Purple	Sky blue	Purple	Anthraquinone
	Brown	Purple	Blue	Purple	Coumarin
		Blue	Brown	Green	Flavonoid
		Purple	Sky blue	Green	Steroid
		Sky blue	Light blue	Yellow	
		Purple	Brown		

*Penicillium* sp. (SCBE-4)	Brown	Purple	Dark quenching spot	Purple	Anthraquinone
	Orange	Light purple	Brown	Purple	Coumarin
	Brown	Sky blue	Sky blue	Green	Isocoumarin
		Blue	Brown	Brown	Steroid
		Purple	Purple		
		Sky blue			
		Purple			

*Colletotrichum* sp. (SCBE-7)	Brown	Purple	Purple	Brown	Steroid flavonoid
	Brown	Green	Dark purple	Yellow	
	Green	Light purple	Brown	Brown	
		Purple	Purple		

*Pestalotiopsis* sp. (SCBE-3)	Brown	Purple	Brown	Purple	Steroid coumarin
		Blue	Dark quenching spot	Purple	Isocoumarin
		Sky blue	Sky blue	Sky blue	
		Purple	Brown	Brown	
		Purple	Brown	Brown	

*Phyllosistica* sp. (SCLE-7)	Brown	Sky blue	Yellow	Purple	Steroid flavonoid coumarin
		Light blue	Dark quenching spot	Purple	Isocoumarin
		Purple	Blue	Green	
		Purple	Brown	Brown	

*Fusarium* sp. (SCLE-8)	Dark Brown	Blue	Yellow	Purple	Flavonoid isocoumarin
	Blackish brown	Blue	Dark quenching spot	Green	Coumarin
		Sky blue	Blue	Purple	
		Purple	Brown	Dark green	
		Dark blue		Brown	
		Dark blue		Dark Brown	

*Colletotrichum* sp. (SCLE-9)	Yellow	Sky blue	Yellow	Purple	Flavonoid steroid
	Brown	Purple	Dark quenching spot	Purple	
			Brown	Green	
			Sky blue	Brown	
			Yellow		
			Brown		
			Brown		

Leaf	Bottle green	Bottle green	Red	Blue	Coumarin
	Green	Light green	Orange	Purple	Isocoumarin
	Light green	Sky blue	Red	Bottle green	Anthraquinone
	Bottle green	Light green	Orange	Green	
	Light green	Bottle green	Red	Light green	
		Light green		Bottle green	
		Purple		Light green	

Bark	Bottle green	Bottle green	Red	Blue	Anthraquinone
	Green	Light green	Orange	Purple	Coumarin
	Light green	Light green	Red	Bottle green	
	Bottle green	Light purple	Orange	Green	
	Light green		Red	Light green	
				Bottle green	
				Light green	

**Table 3:** Antimicrobial activity of plant extracts and endophytic fungal strain.

Sample (100 *μ*g/disc)	*Diameter of zone of inhibition (mm) (mean* *±* *SD)*				
	*Bacterial strain*				Fungal strain
	*Gram positive*		*Gram negative*		*A*. *Niger*
	*B*. *megatorium*	*S*. *aureus*	*S*. *typhi*	*E*. *coli*	
Bark	—	—	10 ± 0.5	—	18 ± 1.5
SCBE-1	—	—	—	—	—
SCBE-2	—	—	—	—	—
SCBE-3	7 ± 1	7 ± 1.5	8 ± 1.5	10 ± 1	
SCBE-4	10 ± 0.2	15 ± 0.4	16 ± 0.8	15 ± 0.5	—
SCBE-7	—	—	—	—	—
Leaf	13 ± 1	7 ± 1.3	15 ± 1.5	18 ± 0.4	22 ± 0.5
SCLE-7	—	—	—	—	—
SCLE-8	—	—	—	—	—
SCLE-9	—	7 ± 1	—	—	—
Kanamycin (30 *μ*g/disc)	20 ± 0.5	30 ± 1	25 ± 0.2	28 ± 0.5	nd
Ketokonazole (30 *μ*g/disc)	nd	nd	nd	nd	18 ± 1

“—” indicates no activity; “nd” indicates not done.

## Data Availability

The data used to support the findings of this study are available from the corresponding author upon request.

## References

[1] Yenn T., Ibrahim D. Endophytic fungi from *Syzygium cumini* (L.) skeels leaves and its potential as antimicrobial agents.

[2] Strobel G., Daisy B., Castillo U., Harper J. (2004). Natural products from endophytic microorganisms. *Journal of Natural Products*.

[3] Mustafa S., Betul O., Celal B., Zeliha S. (2021). Bioactivity of EtOH and MeOH extracts of basidiomycetes mushroom stereum hirsutum on atherosclerosis. *Archives of Razi Institute*.

[4] Sevindik M., Akgul H., Selamoglu Z., Braidy N. (2020). Antioxidant and antigenotoxic potential of infundibulicybe geotropa mushroom collected from north western Turkey. *Oxidative Medicine and Cellular Longevity*.

[5] Sevindik M., Özdemir B., Braidy N., Akgül H., Akata I., Selamoglu Z. (2021). Potential cardiogenic effects of poisonous mushrooms. *The Journal of Fungus*.

[6] Bal C., Baba H., Akata I., Sevindik M., Selamoğlu Z., Akgül H. (2022). Biological activities of wild poisonous mushroom entoloma sinuatum (boletales). *KSU Journal of Agriculture and Nature*.

[7] Treasure U. N., Christiana A. C., Maduabuchi E. P. (2020). The isolation, identification and antimicrobial activities of endophytic fungi from *Azadirachta indica*. *GSC Biological and Pharmaceutical Sciences*.

[8] Jain P., Sharma P. (2012). Probiotics and their efficacy in improving oral health: a review. *Journal of Applied Pharmaceutical Science*.

[9] Wilson D. (1995). Endophyte: the evolution of a term, and clarification of its use and definition. *Oikos*.

[10] Tolulope R., Adeyemi A., Erute M., Abiodun T. (2015). Isolation and screening of endophytic fungi from three plants used in traditional medicine in Nigeria for antimicrobial activity. *International Journal of Green Pharmacy*.

[11] Strobel G., Daisy B. (2003). Bioprospecting for microbial endophytes and their natural products. *Microbiology and Molecular Biology Reviews*.

[12] Berdy J. (2005). Bioactive microbial metabolites. *Journal of Antibiotics*.

[13] Chagas V. T., França L. M., Malik S., Paes A. M. D. A. (2015). *Syzygium cumini* (L.) skeels: a prominent source of bioactive molecules against cardiometabolic diseases. *Frontiers in Pharmacology*.

[14] Yadav M., Yadav A., Yadav J. P. (2014). In vitro antioxidant activity and total phenolic content of endophytic fungi isolated from *Eugenia jambolana* lam. *Asian Pacific Journal of Tropical Medicine*.

[15] Jain P., Sharma P. (2015). Antagonistic activity of endophytic fungi isolated from *Syzygium cumini* (L.) skeels. *International Journal of Library Science and Research*.

[16] Kusari S., Lamshöft M., Zühlke S., Spiteller M. (2008). An endophytic fungus from *Hypericum perforatum* that produces hypericin. *Journal of Natural Products*.

[17] Chowdhury N. S., Sohrab M. H., Rony S. R. (2016). Identification and bioactive potential of endophytic fungi from *Monochoria hastata* (L.) solms. Bangladesh. *Journal of Botany*.

[18] Bhakuni D. S., Dhar M., Dhar M., Dhawan B., Mehrotra B. (1969). Screening of Indian plants for biological activity: part II. *Indian Journal of Experimental Biology*.

[19] Sadananda T., Govindappa M., Ramachandra Y. (2014). In vitro antioxidant activity of lectin from different endophytic fungi of *Viscum album* L. *British Journal of Pharmaceutical Research*.

[20] Devi N., Prabakaran J. (2014). Bioactive metabolites from an endophytic fungus penicillium sp. isolated from *Centella asiatica*. *Current Research in Environmental & Applied Mycology*.

[21] Crouch J. A., Clarke B. B., Hillman B. I. (2005). Phylogenetic relationships and fungicide sensitivities of *Colletotrichum graminicola* isolates from turfgrass in North America. *International Turfgrass Society Research Journal*.

[22] White T. J., Bruns T., Lee S., Taylor J. (1990). Amplification and direct sequencing of fungal ribosomal RNA genes for phylogenetics. *PCR protocols: A Guide to Methods and Applications*.

[23] Alzoreky N., Nakahara K. (2003). Antibacterial activity of extracts from some edible plants commonly consumed in Asia. *International Journal of Food Microbiology*.

[24] Saral Ö., Yildiz O., Aliyazicioğlu R. (2016). Apitherapy products enhance the recovery of CCL4-induced hepatic damages in rats. *Turkish Journal of Medical Sciences*.

[25] Rechenchoski D. Z., Dambrozio A. M. L., Vivan A. C. P. (2017). Antimicrobial activity evaluation and comparison of methods of susceptibility for *Klebsiella pneumoniae* carbapenemase (KPC)-producing *Enterobacter spp*. isolates. *Brazilian Journal of Microbiology*.

[26] Hannana S., Afroz F., Begum M. (2020). Bioactive potential of endophytic fungi isolated from *Phyllanthus niruri* L.. *Bangladesh Journal of Scientific & Industrial Research*.

[27] Sushanto G., Gitishree D., Sandeep K. S., Han-Seung S., Kumar P. J. (2016). Endophytes: a treasure house of bioactive compounds of medicinal importance. *Frontiers in Microbiology*.

[28] Tiwari P., Bae H. (2022). Endophytic fungi: key insights, emerging prospects, and challenges in natural product drug discovery. *Microorganisms*.

[29] Guarnaccia V., Groenewald J. Z., Woodhall J. (2018). Diaporthe diversity and pathogenicity revealed from a broad survey of grapevine diseases in Europe. *Persoonia: Molecular Phylogeny and Evolution of Fungi*.

[30] Xu T. C., Lu Y. H., Wang J. F. (2021). Bioactive secondary metabolites of the genus diaporthe and anamorph phomopsis from terrestrial and marine habitats and endophytes: 2010–2019. *Microorganisms*.

[31] Kim J. W., Shim S. H. (2019). The fungus colletotrichum as a source for bioactive secondary metabolites. *Archives of Pharmacal Research*.

[32] Rios J.-L., Recio M. C., Villar A. (1988). Screening methods for natural products with antimicrobial activity: a review of the literature. *Journal of Ethnopharmacology*.

[33] Zhao J., Li C., Wang W. (2013). Hypocrea lixii, novel endophytic fungi producing anticancer agent cajanol, isolated from pigeon pea (C. ajanus cajan (L.) M. illsp.). *Journal of Applied Microbiology*.

[34] Guo D.-L., Qiu L., Feng D. (2020). Three new *α*-pyrone derivatives induced by chemical epigenetic manipulation of *Penicillium herquei*, an endophytic fungus isolated from *Cordyceps sinensis*. *Natural Product Research*.

[35] Harper J. K., Arif A. M., Ford E. J. (2003). Pestacin: a 1,3-dihydro isobenzofuran from *Pestalotiopsis microspora* possessing antioxidant and antimycotic activities. *Tetrahedron*.

[36] Wikee S., Lombard L., Crous P. W. (2013). Phyllosticta capitalensis, a widespread endophyte of plants. *Fungal Diversity*.

[37] de Lamo F. J., Takken F. L. W. (2020). Biocontrol by *Fusarium oxysporum* using endophyte-mediated resistance. *Frontiers of Plant Science*.

[38] Kaczorowski G. J., Garcia M. L., Bode J., Hess S. D., Patel U. A. (2011). The importance of being profiled: improving drug candidate safety and efficacy using ion channel profiling. *Frontiers in Pharmacology*.

[39] Zhao J., Sun W., Shan T. (2012). Antimicrobial metabolites from the endophytic fungus *Gliomastix murorum* Ppf8 associated with the medicinal plant paris polyphylla var. yunnanensis. *Journal of Medicinal Plants Research*.

[40] Liu X., Dong M., Chen X., Jiang M., Lv X., Yan G. (2007). Antioxidant activity and phenolics of an endophytic *Xylaria sp*. from *Ginkgo biloba*. *Food Chemistry*.

[41] Song Y. C., Huang W. Y., Sun C., Wang F. W., Tan R. X. (2005). Characterization of graphislactone A as the antioxidant and free radical-scavenging substance from the culture of *Cephalosporium sp*. IFB-E001, an endophytic fungus in Trachelospermum jasminoides. *Biological and Pharmaceutical Bulletin*.

[42] Wang L.-W., Xu B.-G., Wang J.-Y. (2012). Bioactive metabolites from *Phoma* species, an endophytic fungus from the Chinese medicinal plant *Arisaema erubescens*. *Applied Microbiology and Biotechnology*.

[43] Chowdhury N. S., Sohrab M. H., Rana M. S., Hasan C. M., Jamshidi S., Rahman K. M. (2017). Cytotoxic naphthoquinone and azaanthraquinone derivatives from an endophytic *Fusarium solani*. *Journal of Natural Products*.

[44] Khan N., Afroz F., Begum M. N. (2018). Endophytic fusarium solani: a rich source of cytotoxic and antimicrobial napthaquinone and aza-anthraquinone derivatives. *Toxicology Reports*.

[45] Tungmunnithum D., Thongboonyou A., Pholboon A., Yangsabai A. (2018). Flavonoids and other phenolic compounds from medicinal plants for pharmaceutical and medical aspects: an overview. *Medicine (Baltimore)*.

